# Efficient Photocatalytic Activities of TiO_2_ Hollow Fibers with Mixed Phases and Mesoporous Walls

**DOI:** 10.1038/srep15228

**Published:** 2015-10-15

**Authors:** Huilin Hou, Minghui Shang, Lin Wang, Wenge Li, Bin Tang, Weiyou Yang

**Affiliations:** 1 Research Institute of Surface Engineering, Taiyuan University of Technology, Taiyuan City, 030024, P.R. China; 2 Institute of Materials, Ningbo University of Technology, Ningbo City, 315016, P.R. China; 3 Merchant Marine College, Shanghai Maritime University, Shanghai City, 201306, P.R. China

## Abstract

Currently, Degussa P25, with the typical mixed phases of anatase and rutile TiO_2_, is widely applied as the commercial photocatalysts. However, there are still some of obstacles for the P25 nanoparticles with totally high photocatalytic activities, especially for the catalytic stability due to their inevitable aggregation of the nanoparticles when used as the photocatalysts. In the present work, we reported the exploration of a novel TiO_2_ photocatalyst, which could offer an ideal platform for synergetic combination of the mixed-phase composition, hollow architecture and mesoporous walls for the desired excellent photocatalytic efficiency and robust stability. The mesoporous TiO_2_ hollow nanofibers were fabricated via a facile single capillary electrospinning technique, in which the foaming agents were used for creating mesopores throughout the walls of the hollow fibers. The obtained hollow fibers exhibit a high purity and possess the mixed phases of 94.6% anatase and 5.4% rutile TiO2. As compared to P25, the as-fabricated mesoporous TiO2 hollow fibers exhibited much higher efficient photocatalytic activities and stabilities toward the hydrogen evolution with a rate of ~499.1 μmol g^−1^·h^−1^ and ~99.5% degradation Rhodamine B (RhB) in 60 min, suggesting their promising application in efficient photocatalysts.

Semiconductor photocatalyst materials are extensively explored for the solar energy utilization, which offer a possible strategy to address the environmental problem and energy crisis^1^,^2^. Up to date, numerous interests have been focused on the titanium dioxide (TiO_2_) material, due to its low cost, ready commercial availability, and long-term stability against the photochemical corrosion in aggressive aqueous environments^3^,^4^.

Generally, TiO_2_ has four typical allotropic forms including anatase, brookite, rutile, and TiO_2_(B), among which the anatase has been considered as the one with the best photoactivity^5^. Compared with the pure TiO_2_ material, the mixed-phase TiO_2_ photocatalysts such as commercial Degussa P25 (composed by ~80% anatase and ~20% rutile) and anatase/TiO_2_(B) fibers, can exhibit much higher photocatalytic activity^6^,^7^,^8^,^9^,^10^,^11^, which could be mainly attributed to the enhanced charge transfer caused by the energy difference between the conduction band (CB) edges of two phases. In addition to the TiO_2_ crystallinity, porous structure of the photocatalyst is beneficial for the adsorption of the reactants, and thus can significantly improve the photocatalytic efficiencies. For example, one-dimensional (1D) TiO_2_ hollow structures are of great interest in the viewpoint of their potential various applications and fundamental phenomena specific to a confined nanostructure^12^,^13^,^14^,^15^,^16^,^17^. Particularly, the hollow TiO_2_ fibers with designed mesoporous walls have received remarkable attention owing to their unique hierarchical pore structure, which is helpful for improved capabilities of mass transport through the material body and maintenance of a specific surface area on the level of a satisfactory porosity^18^,^19^,^20^,^21^,^22^. That is to say, the efficient photocatalytic activities of the TiO_2_ semiconductors could be expected, once they are grown into the fiber structures together with hollow bodies, mixed phases and mesoporous walls. However, the reported works often suffered from tedious procedures or special conditions. Thereby, it is still a challenge for developing a simple and facile route to generate mesoporous TiO_2_ hollow nanofibers.

In the present work, we report the exploration of a novel TiO_2_ photocatalyst, which could offer an ideal platform for synergetic combination of the mixed-phase composition, hollow architecture and mesoporous walls for the desired excellent photocatalytic efficiency and long-term stability. The mixed-phase anatase/rutile TiO_2_ hollow fibers are fabricated via a simple electronspinning method. As illustrated in Fig. 1(a), the hollow structures are obtained by removing the inside paraffin oil during the air calcination, and the foaming agents (DIPA) are concomitantly decomposed into abundant vapor phases to create the thoroughly mesoporous walls. As expected, the obtained hollow fibers, with the mixed phases of anatase and rutile TiO_2_ as well as mesoporous walls exhibit excellent photocatalytic performances in hydrogen production and dye degradation under UV light irradiation.

## Methods

### Raw materials

Polyvinylpyrrolidone (PVP, *M*_*W*_ ≈ 1300000), butyl titanate (TBOT), diisopropyl azodiformate (DIPA), paraffin oil, absolute ethyl alcohol, acetic acid, deionized water and rhodamine B (RhB) were purchased from Aladdin (Shanghai, China). All materials were directly used as received without further treatment.

### Preparation of msoporous TiO_2_ hollow fibers

In a typical experimental procedure, 0.6 g of PVP, 3.0 g of TBOT and 0.5g of DIPA were firstly dissolved in 7 mL absolute ethyl alcohol with stirring vigorously for 2 h. Then, 0.5 g of CTAB and 2 ml paraffin oil were added into the above solution followed by being subjected to the magnetic stirring further for 5 h. Subsequently, the above precursor microemulsions were transformed into a plastic syringe with a stainless steel nozzle (anode, diameter: 0.2 mm). The tip of the stainless steel nozzle was placed in the front of a metal cathode (collector) with a fixed distance of 20 cm between the nozzle and the collector. An electrical potential of 18 kV and a flow rate of 1 mL h^−1^ were applied for electrospinning precursor fibers. The as-spun polymer fibers were dried in an oven at 60 °C, followed by being located in a quartz crucible and placed at the center of a conventional tube furnace. Finally, the precursor fibers were heated up to the desired temperature of 500 °C with a heating rate of 1 °C min^−1^, and maintained there for 3 h in air, followed by furnace-cool to ambient temperature.

### Structure characterization

The obtained products were characterized with X-ray powder diffraction (XRD, D8 Advance, Bruker, Germany) with Cu K*α* radiation (λ = 1.5406 Å), field emission scanning electron microscopy (FESEM, S-4800, Hitachi, Japan), and high-resolution transmission electron microscopy (HRTEM, JEM-2010F, JEOL, Japan) equipped with energy dispersive X-ray spectroscopy (EDX, Quantax-STEM, Bruker, Germany). The porous properties of the as-prepared mesoporous fibers were characterized using N_2_ adsorption at −195.8 °C on a specific surface area and porosity analyzer (ASAP 2020HD88, Micromeritics, USA).

### Photocatalytic activity measurements

The photocatalytic activities of the resultant products were firstly evaluated for hydrogen evolution. The photocatalytic reaction is performed in an inner-irradiation quartz annular reactor with a 300 W Xenon lamp (CEL, HUL300), a vacuum pump, a gas collection, a recirculation pump and a water-cooled condenser. The as-synthesized samples (0.1 g) were suspended in deionized water and methanol mixed solutions (40 mL, 3:1) by an ultrasonic oscillator, respectively. Then the mixture was transferred into the reactor and deaerated by the vacuum pump. The Xenon lamp was utilized as a light source, and the cooling water was circulated through a cylindrical Pyrex jacket located around the light source to maintain the reaction temperature. The reactor was sealed with ambient air during irradiation, and the hydrogen evolution were monitored by an online gas chromatography (GC, 7900) equipped with a Porapak-Q column, high-purity nitrogen carrier and a thermal conductivity detector (TCD). In order to investigate the stability and recyclability, the products were re-used in the same reaction for 3 cycles. Furthermore, degradation of rhodamine B (RhB, Aladdin, Shanghai, China) was studied to evaluate its photocatalytic ability. The photocatalytic reaction is performed at an inner-irradiation quartz annular reactor, which has a Xenon lamp (λ > 320 nm, CEL-Hx F300, Beijing, China) and a water-cooled condenser. Typically, 1.2 mg of RhB and 40 mg of the as-prepared catalysts were dispersed in 120 ml deionized water. Prior to irradiation, the suspensions were magnetically stirred in the dark for 60 min to ensure the establishment of an adsorption-desorption equilibrium between the photocatalyst and the RhB dye. An aliquot (3 mL) of the solution was taken at a certain time interval (10 min) during the reaction and analyzed on the UV-visible spectrophotometer as mentioned above. The change in RhB absorbance in the solution was used to monitor the extent of reaction at the given irradiation time intervals. For comparison, Degussa P25 was commercially available, and used directly for the photocatalytic activity for H_2_ generation and degradation of RhB.

## Results

**Figure 1:**
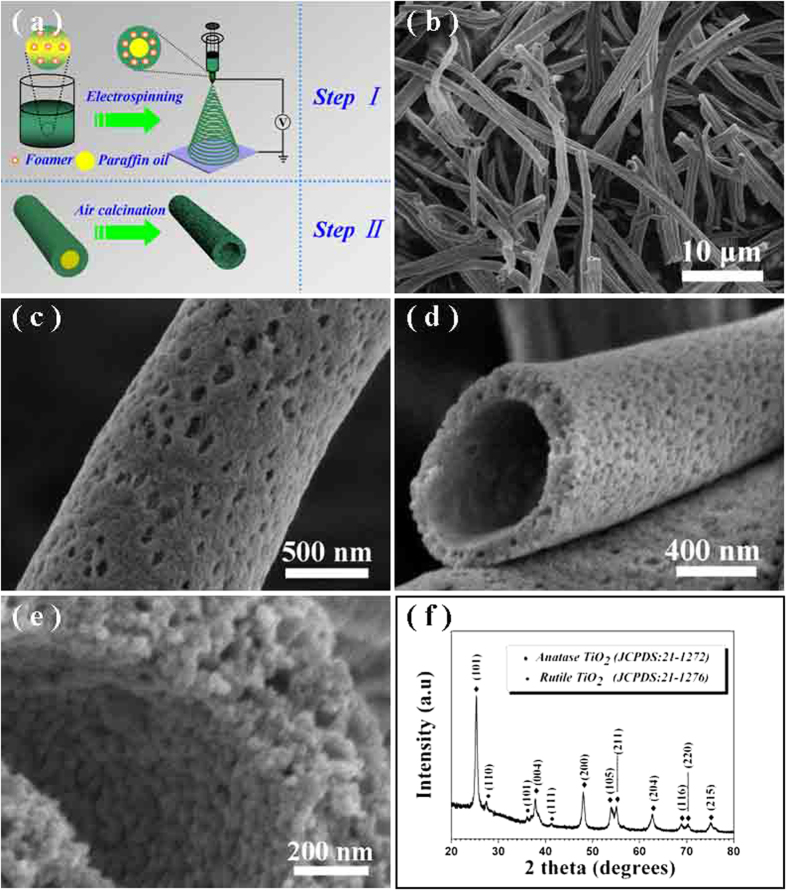
(**a**) Schematic illustration for the formation of mesoporous TiO_2_ hollow fibers via the foaming assisted electrospinning. (**b**) A typical SEM image of the calcined products under a low magnification. (**c**–**e**) Typical SEM images of the calcined products under higher magnifications and different views. (**f**) A representative XRD pattern of the calcined products.

SEM was firstly employed to meticulously study the morphology and microstructure of the precursor fibers and their corresponding calcined products. The resultant precursor fibers (Figure S1 (a), Supplementary Information) are continuous with the diameters in the range of 1 ∼ 1.5 μm and up to several hundred of millimeters in length. Figure 1(b–e) are the typical SEM images under different magnifications and views of the corresponding calcined products. It seems that the long continuous precursor fibers have been converted into hollow structures with the diameters reduced to ~900 nm, which could be mainly ascribed to the elimination of the organisms during the air calcination process (see Fig. 1(b)). Figure 1(c–e) show the interior and external surface view under different magnifications of the TiO_2_ hollow fibers, respectively, disclosing that numerous pores exist on the fiber surface and the hollow fiber walls are thoroughly mesoporous. The formation of the hollow and mesoporous structures could be mainly attributed to the decomposition and removing of organics existed within the as-spun polymeric fibers. The powder X-ray diffractograms (XRD) in wide angles are used to characterize the phase compositions of the calcined products. As shown in Fig. 1(f), besides the dominant diffraction peaks of anatase TiO_2_ (JCPDS, No. 21-1272), some weak diffraction peaks of rutile phase (JCPDS, No. 21-1276) can be also clearly detected, suggesting that the obtained products possess the mixed phases of anatase and rutile TiO_2_. According to the XRD data, these two phases existed in the fibers can be calculated by using semi-quantitative calculation method, disclosing that the as-synthesized hollow TiO_2_ fibers are composed of 94.6% anatase and 5.4% rutile. As mentioned above, the mixed-phase structure favors the exploration of efficient photocatalysts, since the photogenerated charge transfer between the involved two phases can greatly inhibit the charge recombination of the holes/electrons, thus leading to the enhancement of the photocatalytic activities^23^,^24^,^25^.

**Figure 2:**
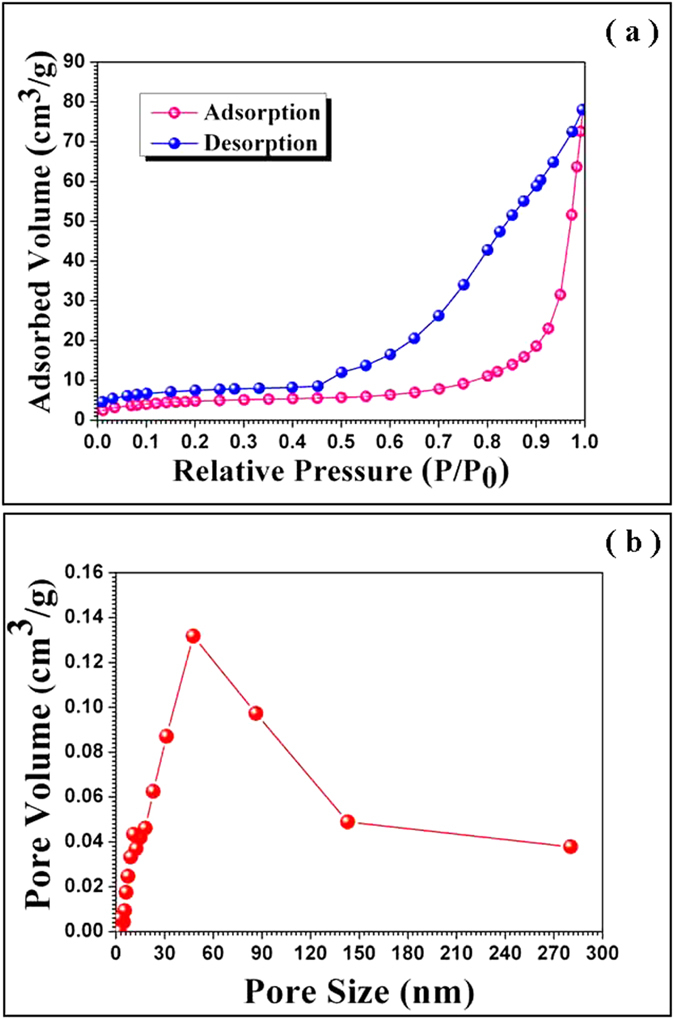
(**a**) Nitrogen adsorption-desorption isotherm curve of mesoporous TiO_2_ hollow nanofibers. (**b**) Pore size distribution curve of the mesoporous TiO_2_ hollow nanofibers.

The nitrogen adsorption measurements (Fig. 2(a) reveal that the as-fabricated products exhibit the type IV isotherm behavior with H3 hysteresis, implying that the obtained hollow fibers are mesoporous with a BET surface area of *~*27.2 m^2^/g. According to the Barrett–Joyner–Halenda (BJH) pore size distribution analysis determined from the adsorption branches (Fig. 2(b)), the average BJH pore diameter is centered at ~38 nm.

**Figure 3:**
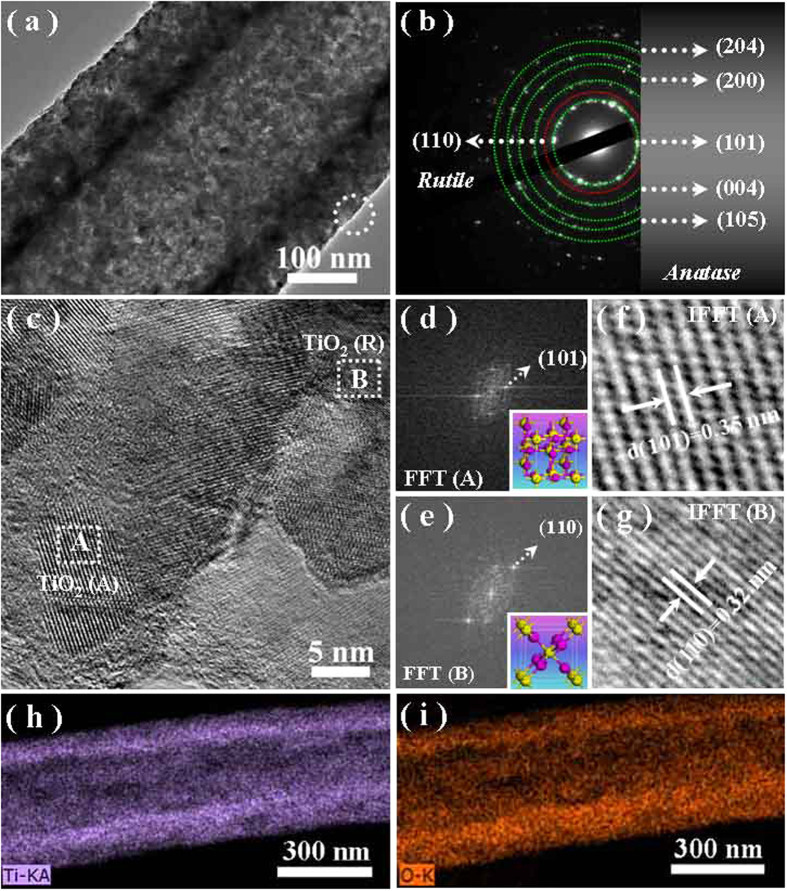
(**a**) A representative TEM image of the mesoporous TiO_2_ hollow fibers. (**b**) The corresponding SAED pattern. (**c**) A representative HRTEM image of the mesoporous hollow fibers recorded from the marked area in (**a**). (**d**,**e**) Fast fourier transformation (FFT) images of the marked areas of A and B in (**c**). The insets are the partial geometry models of anatase and rutile TiO_2_, respectively. (**f**,**g**) The corresponding inverse fast fourier transformation (IFFT) images of (**d**,**e**), respectively. (**h**,**i**) The element mappings of Ti and O within a single nanofiber.

TEM was further used to investigate the structural details of the mixed-phase hollow fiber, as shown in Fig. 3(a). In agreement with the SEM observations, the inner tunnel is clearly observed by the sharp contrast between TiO_2_ mesoporous wall and the hollow interior. The wall thickness and inner diameter are ~100 nm and ~215 nm, respectively, suggesting the large exposed surface of the products. Figure 3(b) presents the corresponding selected area electron diffraction (SAED) pattern recorded from the single fiber in Fig. 3(a), suggesting its polycrystalline nature. The dominant diffraction spot rings could be sequentially indexed to the crystal planes of (101), (004), (105), (200) and (204) of anatase TiO_2_ (JCPDS, No. 21-1272), and the low-intensity diffraction spot ring matches to the (110) plane of rutile TiO_2_ (JCPDS, No. 21-1276). Furthermore, a representative high-resolution TEM (HRTEM) image (Fig. 2(c)) recorded from the marked area in Fig. 3(a) discloses the mixed-phase structures existed within the hollow fiber bodies. Accordingly, the two fast fourier transformation (FFT) images (Fig. 3(d,e)) provide the electron diffraction signals corresponding to the local lattice patterns recorded from the marked areas of A and B in Fig. 3(c), respectively. Their crystalline structure and crystalline face are determined from the distances between the reciprocal lattice points and center point in the FFT images^23^, implying that both squared regions possess the tetragonal characteristic. Hoverer, the measured distance between the highest intensity point and center one is unequal, suggesting the different phases of these two given selected areas, where the marked areas of A and B belong to anatase (the inset of Fig. 3(d)) and rutile (the inset of Fig. 3(e)), respectively. Figure 3(f,g) are the corresponding fast flourier transformation images (IFFT), further confirmed that *d*-spacing of 0.35 nm matches to the (101) plane of anatase (Fig. 3(f)) and that of 0.32 nm corresponds to the (110) plane of rutile (Fig. 3(g)), respectively. This is in good agreement with the XRD diffraction results, confirming that the obtained products should be composed by the mixed-phase of anatase and rutile TiO_2_. The energy-dispersive X-ray (EDX) spectroscopy analyses (typically shown in Figure S2, Supplementary Information), recorded from a number of fibers and different positions along a single one, suggest that the chemical compositions are identical and mainly composed by Ti and O elements. The Cu and C signal should be from the TEM grid used to support the sample. The element mapping images of Ti (Fig. 3(h)) and O (Fig. 3(i)) exhibit a harmonious ravine distribution throughout the fiber body, verifying the hollow nature of the as-fabricated TiO_2_ nanofibers.

**Figure 4:**
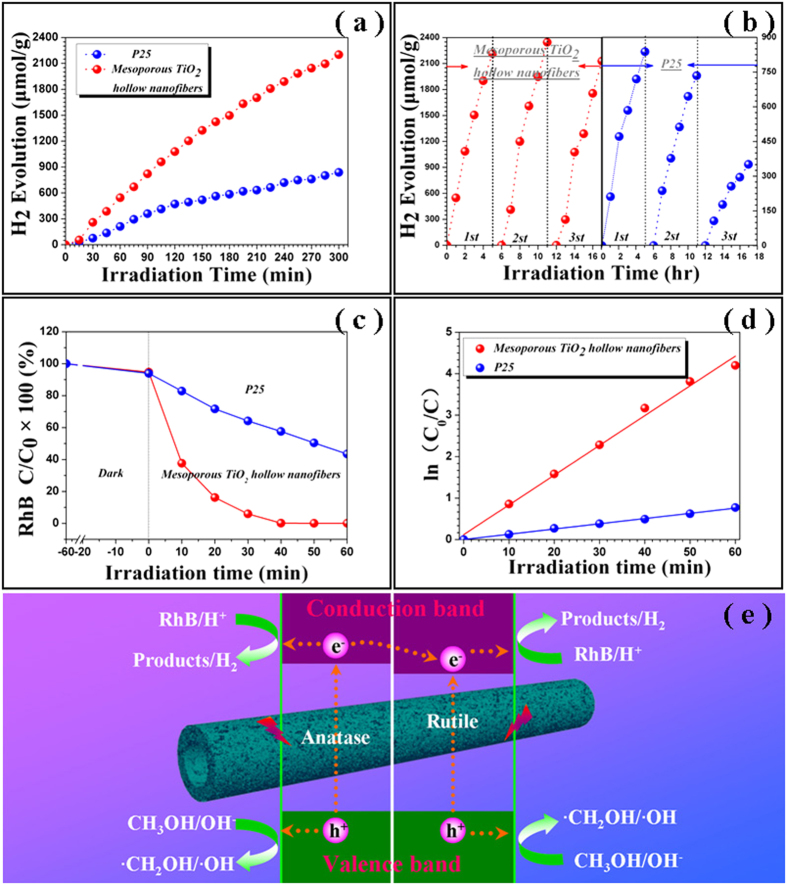
(**a**) The hydrogen production photocatalyzed by the as-fabricated mesoporous TiO_2_ hollow nanofibers as well as p25 under different irradiation times. (**b**) Reusability experiment for photocatalytic H_2_ generation of mesoporous TiO_2_ hollow nanofibers and p25. (**c**) Photocatalytic degradation of RhB (C0 = 10 mg/L) of mesoporous TiO_2_ hollow nanofibers and p25 under UV-visible light irradiation. (**d**) The plot of ln(C0/C) with irradiation time for mesoporous TiO_2_ hollow nanofibers and p25. (**e**) The proposed mechanism for the enhanced photocatalytic activities of the mesoporous TiO_2_ hollow fibers with mixed phases of anatase and rutile.

Photocatalytic evolution of H_2_ on the as-prepared products as well as P25 (the typical SEM image and EDX spectrum, see Figure S3, Supplementary Information) is carried out by using methanol as sacrificial agents and irradiation under a 300 W Xenon arc lamp. Figure 4(a) plots the typical H_2_ evolution kinetic lines over these two photocatalysts. For the mesoporous TiO_2_ hollow fibers, their photocatalytic reactions exhibit a stable H_2_ release rate of ~499.1 μmol g^−1^·h^−1^, which is much higher than that of P25 (*i.e.*, 197.8 μmol g^−1^·h^−1^) and those reported for solid and porous TiO_2_ nanofibers always lower than 400 μmol g^−1^·h^−1^ (see Table 1). In order to investigate their reusability, these two photocatalysts are recovered and re-used for photocatalytic H_2_ production under the identical experimental conditions. As shown in Fig. 4(b), the activity of the mesoporous TiO_2_ hollow fibers is still maintained with no noticeable decrease observed after 3 cycles. However, the photocatalytic ability of P25 has evidently declined after 3 cycles. Obviously, the 1D mesoporous hollow structure exhibits a much better long-term photocatalytic stability than the commercial P25. Additionally, the photocatalytic properties of the as-prepared TiO_2_ hollow nanofibers are evaluated by the decomposition of RhB dye. Figure 4(c) shows their time-dependent degradation of RhB, which are monitored by the maximum band absorbance (also see Figure S4, Supplementary Information), and corrected one by one based on the standard plots of the dye with various RhB concentrations (see Figure S5, Supplementary Information). It seems that, without the light irradiation, the concentration of RhB suspension over the mesoporous TiO_2_ hollow fibers as well as P25 undergoes a tiny change during 60 min magnetic stirring for the sample preparation, suggesting that the dark physics adsorption have little influence on the dye concentration change. The colors of the suspensions catalyzed by the mesoporous TiO_2_ hollow products (the inset in Figure S4(a), Supplementary Information) change gradually from pink to almost colorless, implying the nearly complete photodegradation of RhB. Nevertheless, the suspensions catalyzed by P25 (the inset of Figure S4(b), Supplementary Information) still maintain an obvious light pink color after 60 min illumination, declaring that the some part of the RhB still remained in the suspension without decomposition. The overall degradation efficiency is 99.5% for mesoporous TiO_2_ hollow fibers, which is ~2.5 times to that of P25. To further study the photocatalytic reaction, the pseudo-first-order kinetic model is adopted according to the equation *ln(C*_*0*_*/C)* = *k*, where *C*_*0*_ is the adsorption equilibrium concentration of pollutant before irradiation, *C* is the instantaneous concentration, and *k* is the apparent rate constant with determined reaction time, respectively^5^,^26^. As shown in Fig. 4(d), the corresponding reaction rate constants (*k*) are calculated to be 0.071 and 0.010 min^−1^ for the mesoporous TiO_2_ hollow fibers and P25, respectively. Notably, the present mesoporous hollow products own superior photodegradation efficiency to that of P25. These experimental results verify that, in comparison to the commercial products of P25, the present mesoporous TiO_2_ hollow fibers could be a much higher efficient photocatalyst candidate for both photocatalytic evolution H_2_ production and degradation of hazardous materials.

## Discussion

The growth of mesoporous TiO_2_ hollow fibers could be assigned to follow issues: i) The formation of the hollow interior. As stated in the experimental procedure, the used raw materials of PVP, TBOT, DIPA, absolute ethyl alcohol, CTAB and paraffin oil would cause the formation of microemulsions. As schematically illustrated in Fig. 1(a), the core-shell structure of the as-spun polymeric fibers would be then formed, due to the distinctively different phase interfaces between the mixtures dissolved in the solvent, which cause the formation of a core-shell jet driven by the electrical forces during electrospinning^27^. The core is mainly made up by the trapped paraffin oil, and the shell should be the remained materials of PVP/TBOT/CTAB/DIPA. Once subjected to be calcined at high temperature, the inside paraffin oil core would be completely decomposed into gas phases such as CO_2_ and H_2_O, leading to the formation of the hollow interior of the fiber. ii) The formation of the thoroughly mesoporous walls of the hollow fibers. It should be mainly ascribed to the thermal decomposition of foaming agents of DIPA assembled within the fibers, which would be converted into abundant vapor phases such as CO_2_, NO_2_ and H_2_O^28^, making the creation of mesoporous throughout the walls of the hollow fibers.

To account for the enhanced photocatalytic ability mesoporous TiO_2_ hollow fibers, a tentative mechanism is proposed, as schematically illustrated in Fig. 4(e). It is well known that the crystallite phase and material architecture play a significant role on the photocatalytic performance of TiO_2_ photocatalysts^2^,^3^,^29^. Thus, the excellent photocatalytic performance of mesoporous TiO_2_ hollow fibers might be explained by the following points. i) The mixed phases of anatase and rutile TiO_2_ within the fibers. The present products possess the similar functional mix-phase junction of P25, which leads to the improvement of interfacial charge transfer and effectively limits the charge recombination^20^,^21^,^22^. ii) The well-defined hollow mesoporous nanofibers. The presence of favorable hollow formation with big inner diameters could make the reactant more and easier to contact the photocatalysts, owing to the more active sites offered by the larger exposed surface areas^30^,^31^. iii) The 1D mesoporous architecture walls. This can not only remarkably inhibit the agglomeration of nanoparticles, but also improve the surface adsorption capacity of the reactants. Furthermore, the mesoporous channels existed throughout the fiber bodies also could facilitate the effective transportation of products^32^,^33^. In brief words, the as-fabricated mesoporous TiO_2_ hollow fibers could be considered as the assembly of P25 nanoparticles with the mixed phases of anatase and rutile phases into hollow one-dimensional counterparts with thoroughly mesoporous walls, which offer the platform for synergetic combination of the mix-phase composition, hollow architecture and mesoporous walls to bring a significant enhancement on the photocatalytic activities.

## Conclusions

In summary, we have demonstrated the investigation of mesoporous TiO_2_ hollow nanofiber photocatalysts, which are fabricated via a facile single capillary electrospinning technique^39^,^40^,^41^. The obtained fibers are composed of anatase and rutile mixed phases with well-defined hollow bodies and thoroughly mesoporous walls. The as-fabricated mesoporous TiO_2_ hollow fibers exhibit efficient photocatalytic activities and robust stabilities toward the hydrogen evolution and degradation of RhB, which are ~2.5 times to those of commercial P25, suggesting their promising applications as excellent photocatalysts. It is promising that the mesoporous TiO_2_ hollow nanofibers could offer an ideal platform for synergetic combination of the mixed-phase composition, hollow architecture and mesoporous walls for the desired excellent photocatalytic efficiency and stability, which could provide a novel strategy for the exploration of novel TiO_2_ photocatalysts with high performances.

## Additional Information

**How to cite this article**: Hou, H. *et al.* Efficient Photocatalytic Activities of TiO_2_ Hollow Fibers with Mixed Phases and Mesoporous Walls. *Sci. Rep.* **5**, 15228; doi: 10.1038/srep15228 (2015).

## Supplementary Material

Supplementary Information

## Figures and Tables

**Table 1:** Comparison of the related typical works for H_2_ production using TiO_2_ nanofibers as the Photocatalyst.

Material	Preparation	Morphology	Irradiation conditions	Reaction solution	Activity (μmol g^−1^h^−1^)	Reference
TiO_2_ (B)	hydrothermal	Nanofibers	15 W UV lamp	Neat ethanol	238	34
TiO_2_	Electrospinning	Nanofibers	450 W Hg	Water+MeOH	54	20
TiO_2_ (B)/Pt	hyndrothermal	Nanofibers	15 W UV lamp	Neat ethanol	257	35
TiO_2_/Pt	Electrospinning	Nanofibers	300 W Xe	Water+MeOH	910	36
TiO_2_/Pt	hydrothermal	Nanofibers	300 W Hg	Water+MeOH	310	37
TiO_2_	hydrothermal	Nanofibers	6 UVB lamps	Water +ethanol	30	28
TiO_2_	Electrospinning	Porous Nanofibers	400 W Hg	Water+MeOH	80	17
TiO_2_	Electrospinning	Nanofibers	400 W Hg	Water+AO7	21	38
TiO_2_	Electrospinning	Porous fibers	300 W Xe	Water+MeOH	198	27
TiO_2_	foaming-assisted electrospinning	Mesoporous nanofibers	300 W Xe	Water+MeOH	399	29
TiO_2_	foaming-assisted electrospinning	Mesoporous nanofibers	300 W Xe	Water+MeOH	499	Current work
