# Supplementary material for: Efficient Photocatalytic Activities of TiO2 Hollow Fibers with Mixed Phases and Mesoporous Walls
Source: Sci Rep. 2015 Oct 15;5:15228. doi: 10.1038/srep15228 (PMC4606570; doi:10.1038/srep15228)
Supplement: Supplementary Information [file srep15228-s1.doc]

***Supplementary Information***

**Efficient Photocatalytic Activities of TiO_2_ Hollow Fibers with Mixed Phases and Mesoporous Walls**

*Huilin Hou^1,2^, Minghui Shang^2^, Lin Wang^2^, Wenge Li^3^, Bin Tang^1,*^ and Weiyou Yang^2,*^*

^1^ Research Institute of Surface Engineering, Taiyuan University of Technology, Taiyuan City, 030024, P.R. China.

^2^ Institute of Materials, Ningbo University of Technology, Ningbo City, 315016, P.R. China.

^3^ Merchant Marine College, Shanghai Maritime University, Shanghai City, 201306, P.R. China

***Corresponding author E-mails:*** tangbin@tyut.edu.cn (B. Tang) [weiyouyang@tsinghua.org.cn](mailto:weiyouyang@tsinghua.org.cn) (W. Yang)

***Tel:*** +86-574-87080966

***Fax:*** +86-574-87081221


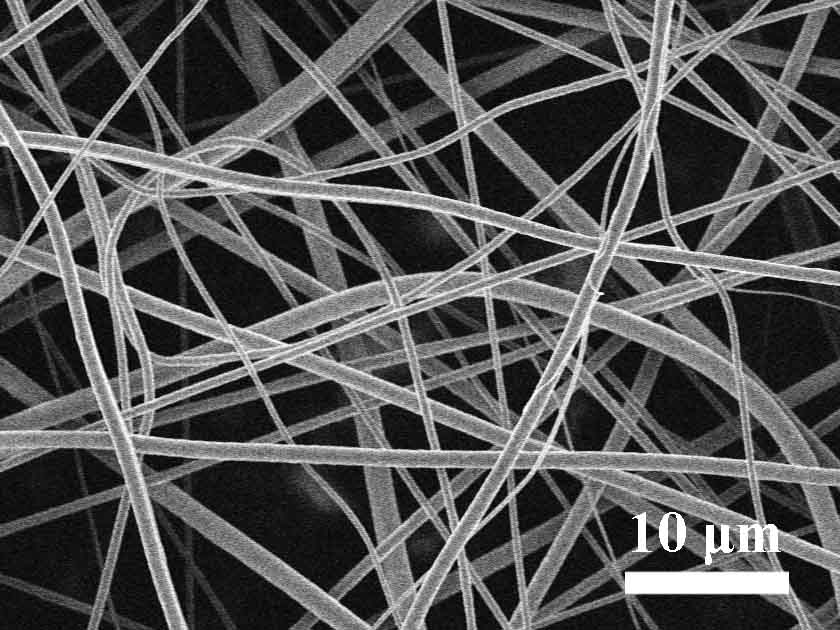


**( a )**


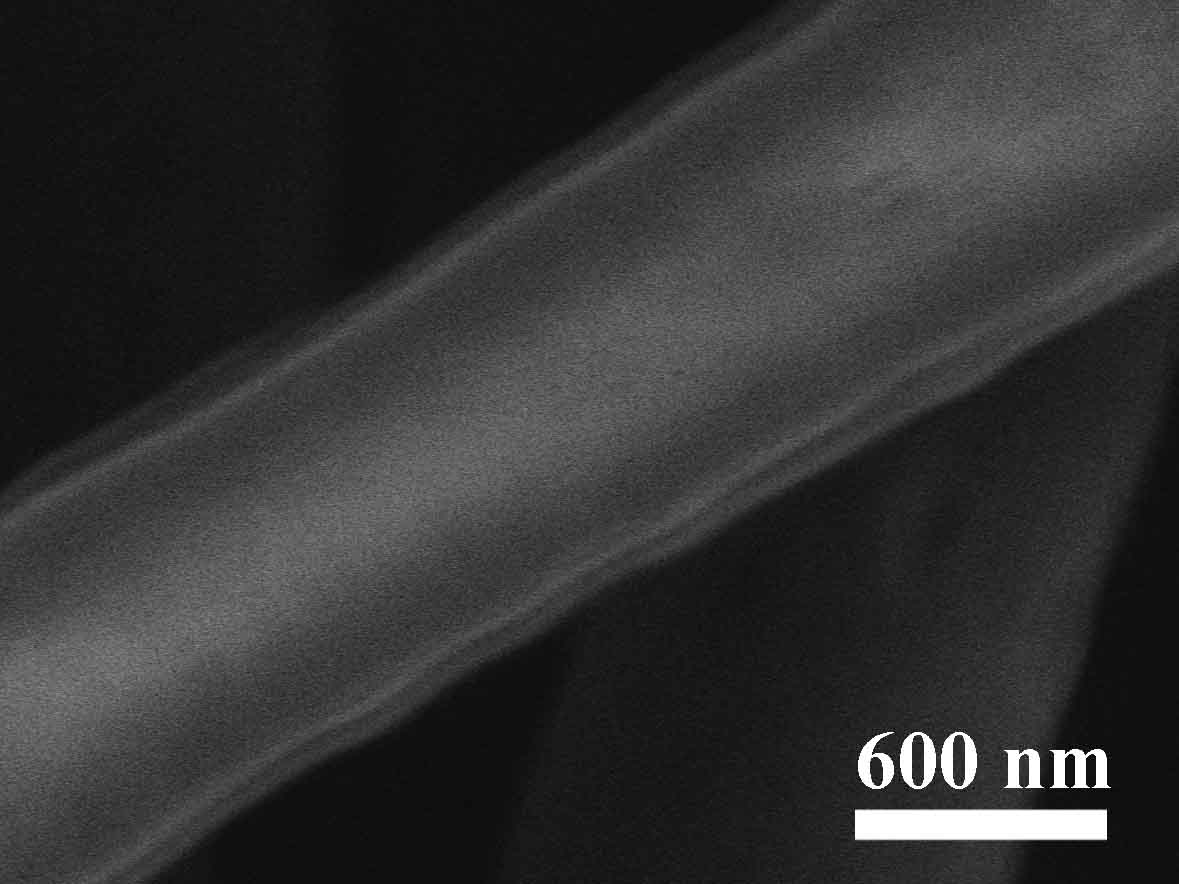


**( b )**

**Figure S1.** Typical SEM images of the as-spun polymeric precursor fibers.


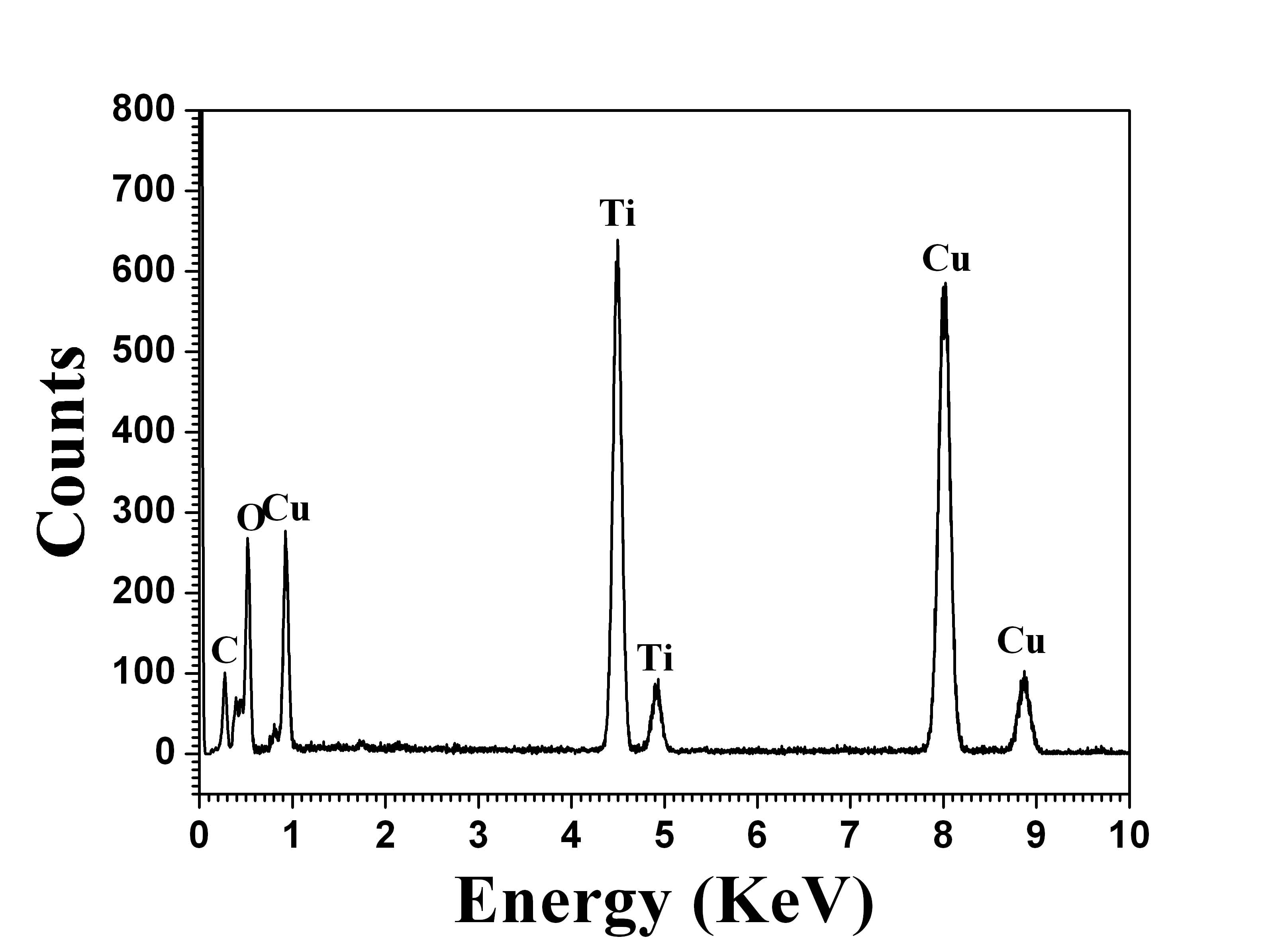


**Figure S2.** A representative EDX spectrum of calcined mesoporous TiO_2_ hollow nanoﬁbers.


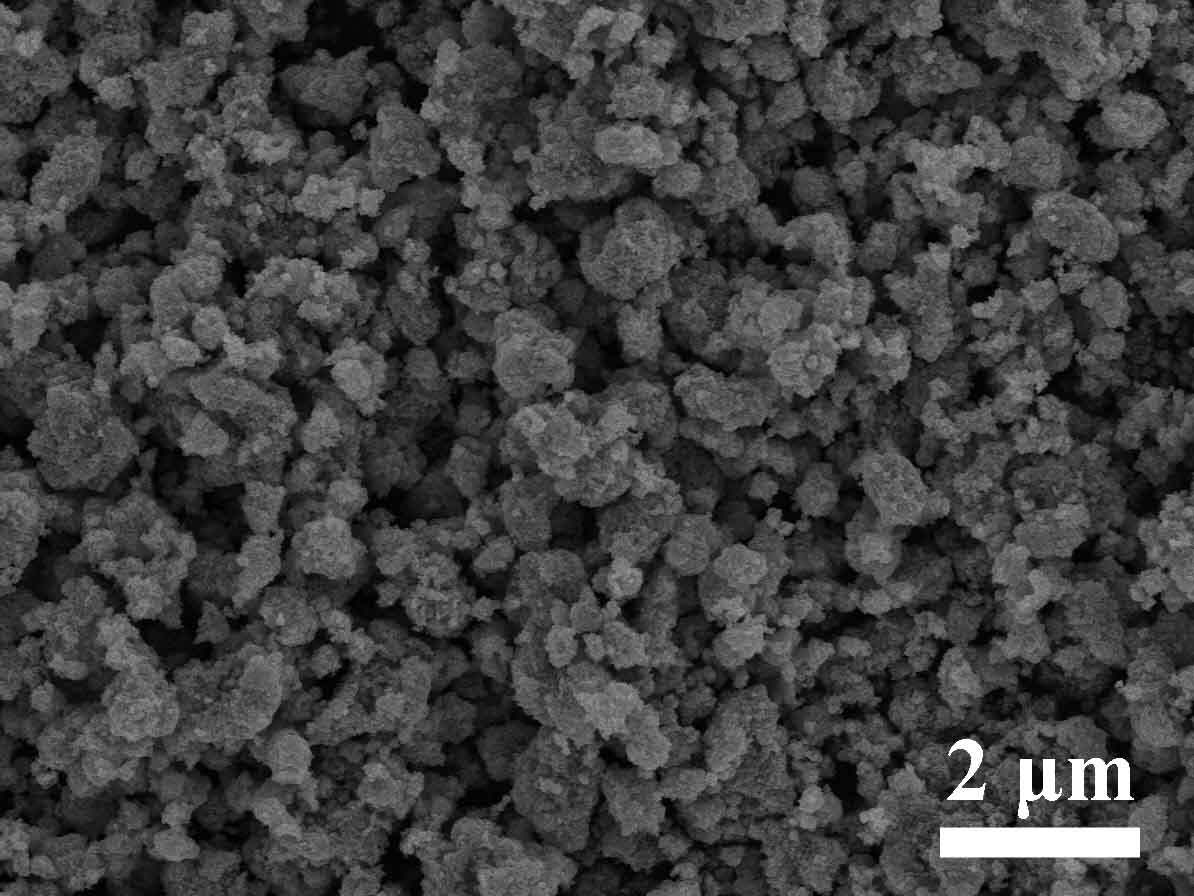


**( a )**


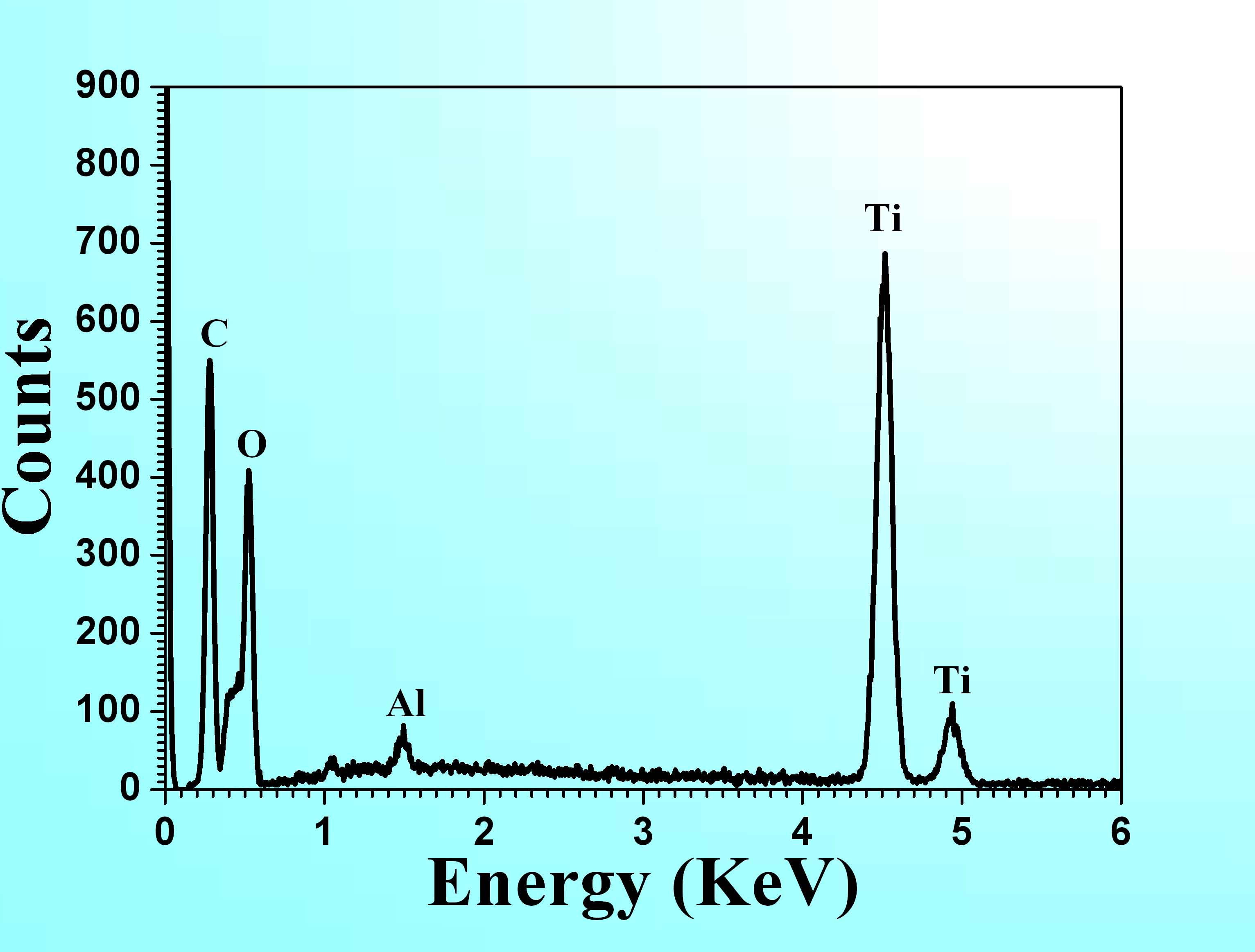


**( b )**

**Figure S3.** (a) A representative SEM image of P25. (b) The corresponding EDX pattern recorded form the marked area in (a).


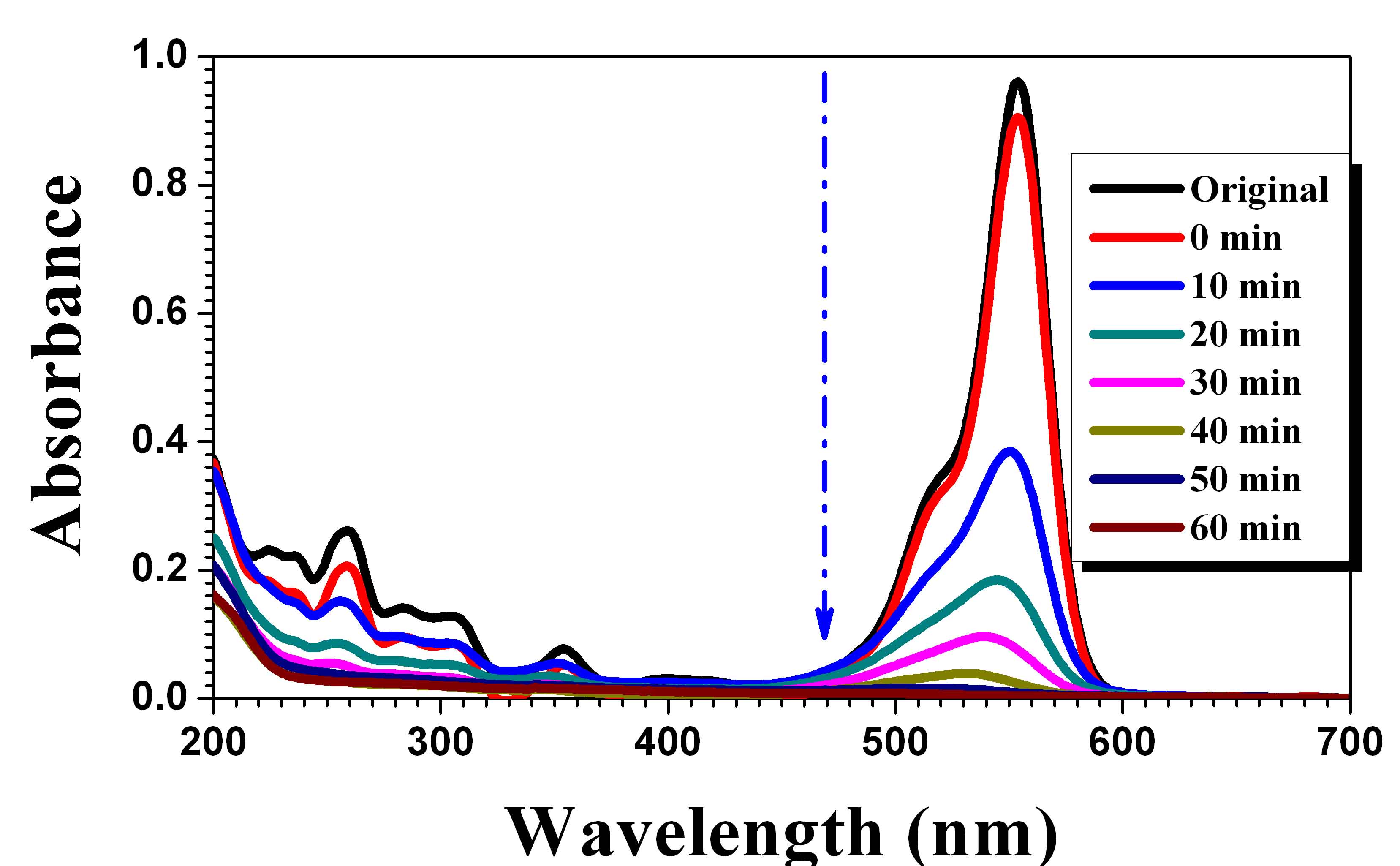

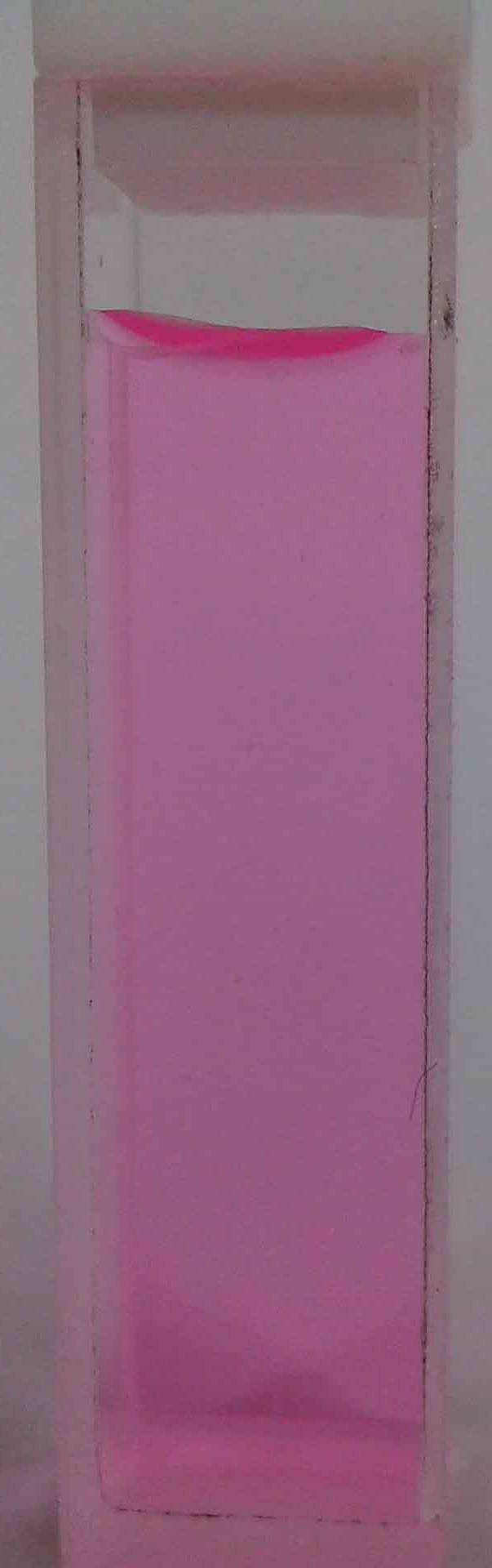

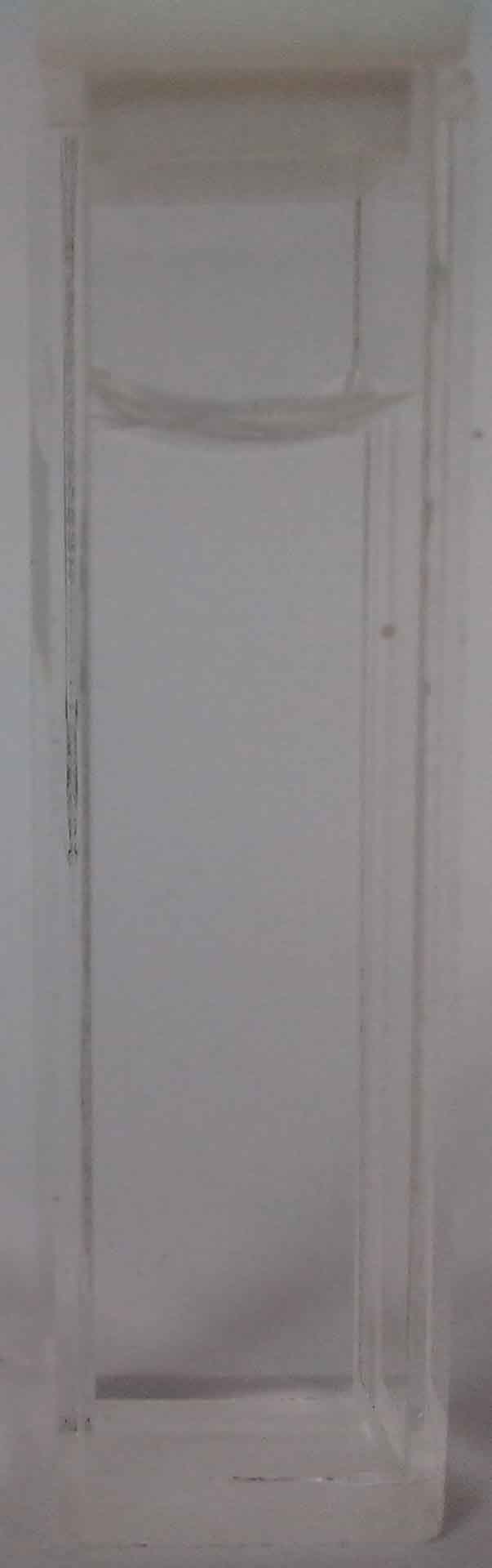


**60 min**

**( a )**

**Mesoporous TiO_2_ hollow fibers**

**( b )**

**P25**


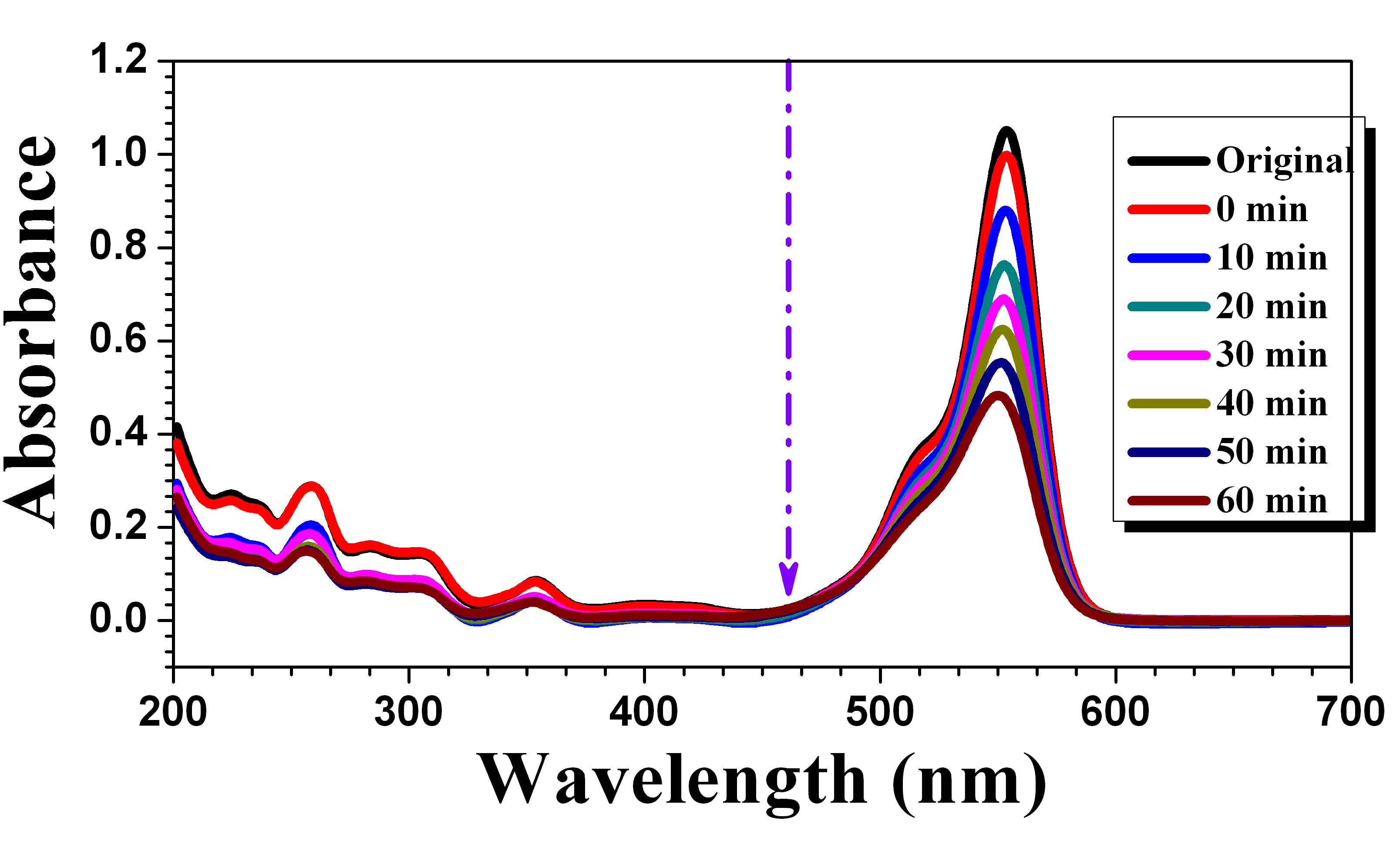


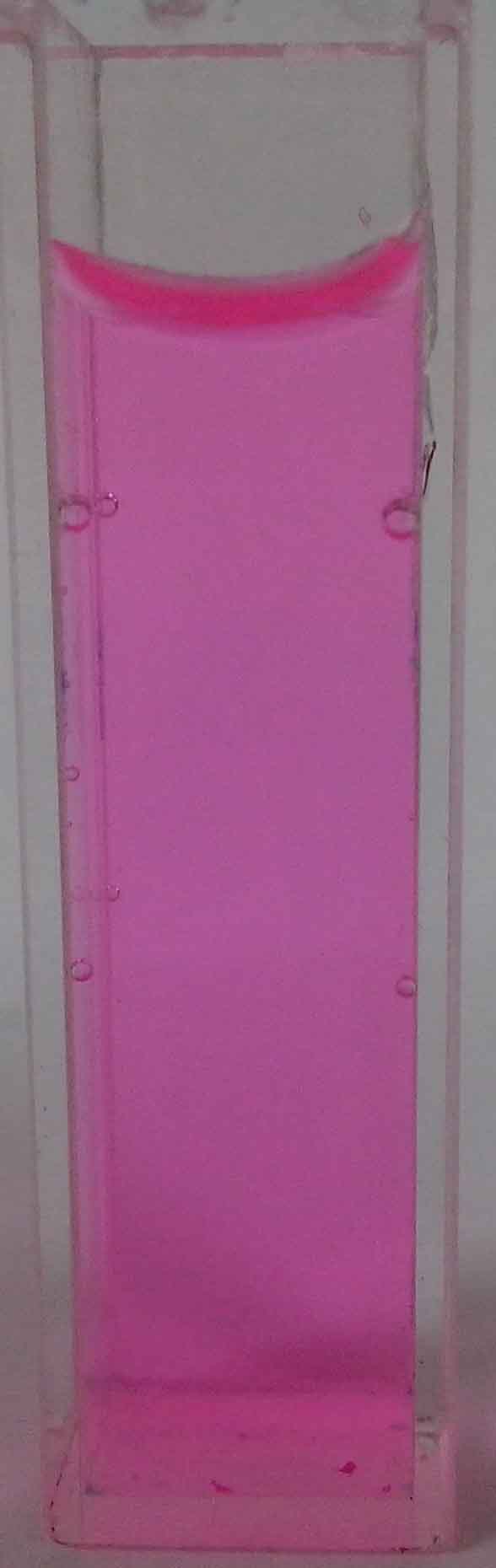

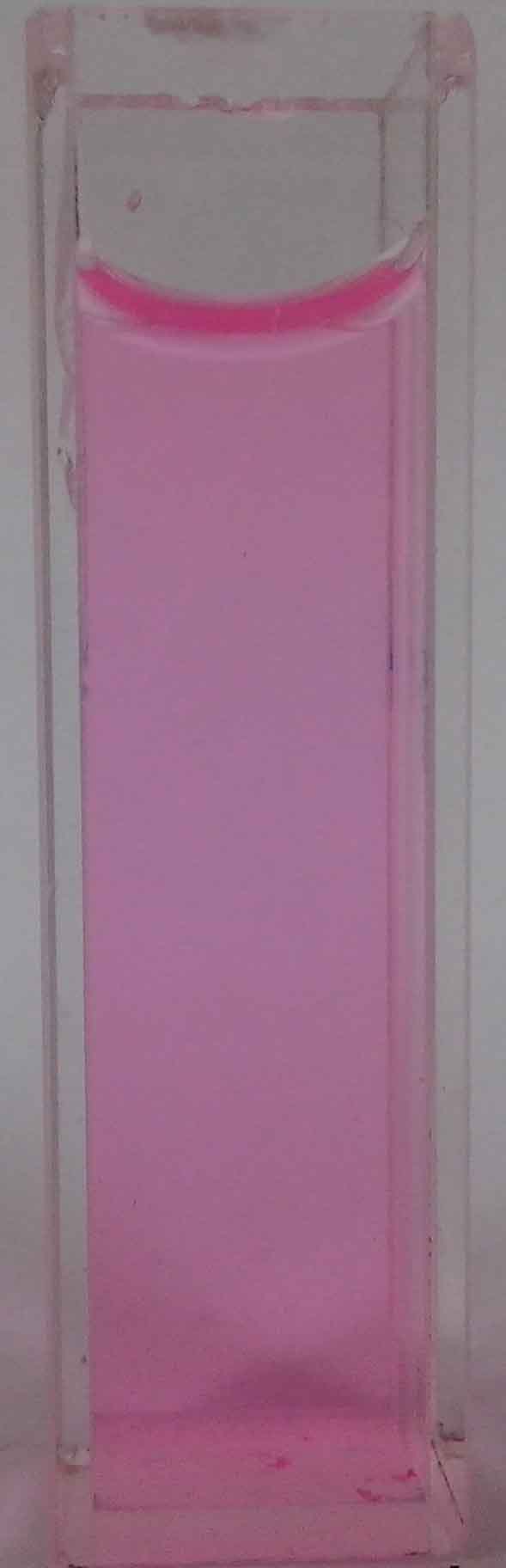


**60 min**

**Figure S4. (a-b)** Temporal UV-visible adsorption spectral changes of the RhB solutions over the mesoporous hollow TiO_2_ fibers and P25. The insets are the corresponding color changes with and without UV light irradiation for 60 min, respectively.


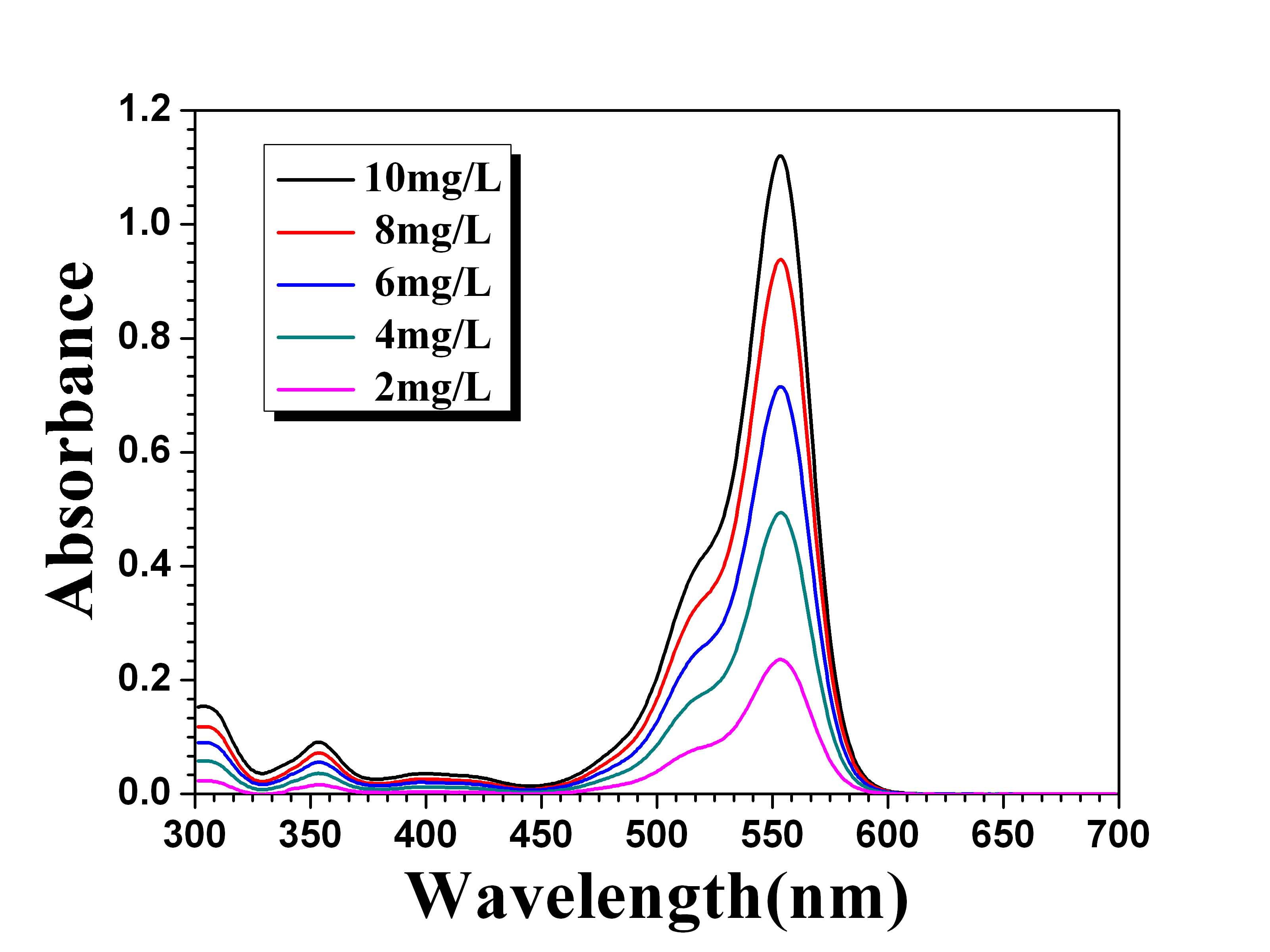

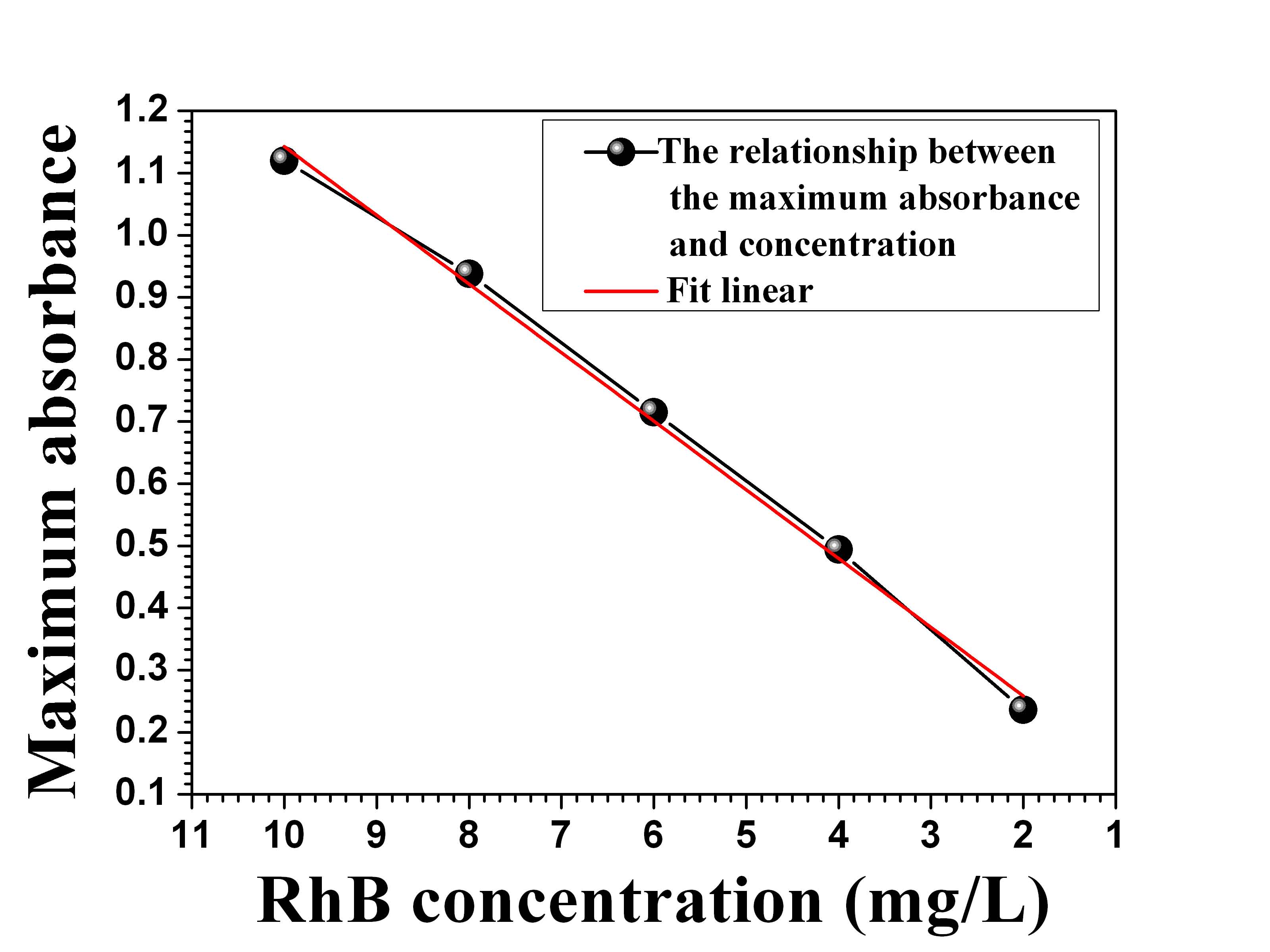


**( a )**

**( b )**


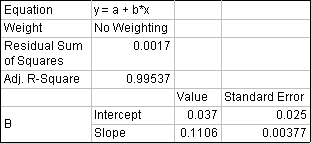


**Figure S5.** (a) Temporal UV-visible adsorption spectralchanges ofthe RhB solutions with different concentrations. (b) The relationship betweenthe maximum absorbanceand concentration. The inset is the linear fitting parameters.
